# Bioavailability for the Improved Therapeutic Profile of *trans*-Dehydrocrotonin Incorporated into a Copaiba Oil Self-Nanoemulsifying Drug Delivery System: Formulation, Physicochemical Characterizations, and Antioxidant In Vitro Effect

**DOI:** 10.3390/ijms26104469

**Published:** 2025-05-08

**Authors:** José Robério de Oliveira Netto, Natália Pignataro Corrêa, Leonardo Bruno Aragão de Araujo, Weslley de Souza Paiva, Hugo Alexandre Oliveira Rocha, Waldenice de Alencar Morais Lima, José Heriberto Oliveira do Nascimento, Daniel Charles dos Santos Macedo, Nereide Stela Santos-Magalhães, Valdir Florêncio da Veiga Júnior, Maria Aparecida Medeiros Maciel

**Affiliations:** 1 Post Graduate Program in Biotechnology (REDE RENORBIO), Federal University of Rio Grande do Norte, Natal 59072-970, RN, Brazil; roberionetto.biotec@gmail.com (J.R.d.O.N.); leobiubao@yahoo.com.br (L.B.A.d.A.); hugo.rocha@ufrn.br (H.A.O.R.); 2 Post Graduate Program in Biotechnology, Potiguar University, Campus Salgado Filho, Natal 59075-000, RN, Brazil; natipignataro96@gmail.com; 3 Department of Biochemistry, Federal University of Rio Grande do Norte, Natal 59072-970, RN, Brazil; wdspaiva@gmail.com; 4 Department of Pharmacy, Federal University of Rio Grande do Norte, Natal 59072-970, RN, Brazil; 5 Post Graduate Program in Chemical Engineering, Federal University of Rio Grande do Norte, Natal 59072-970, RN, Brazil; heriberto.nascimento@ufrn.br; 6 Institute Keizo-Asami (LIKA), Federal University of Pernambuco, Recife 50670-901, PE, Brazil; danielcsmacedo92@gmail.com (D.C.d.S.M.); nereide.magalhaes@ufpe.br (N.S.S.-M.); 7 IME-Chemistry Section, Military Institute of Engineering, Rio de Janeiro 22290-270, RJ, Brazil; valdir.veiga@gmail.com

**Keywords:** copaiba oil, 19-*nor*-clerodane *trans*-dehydrocrotonin, SNEDDS system, physicochemical characterizations, antioxidant activity

## Abstract

*Croton cajucara* Benth and *Copaifera reticulata* Ducke are prominent species in the traditional medicine of the Amazon region of Brazil. *Copaifera* species produce oil resin rich in bioactive diterpenes, and *C. cajucara* is a prolific producer of the diterpene 19-*nor*-clerodane *trans*-dehydrocrotonin (*t*-DCTN). This research aimed to develop a self-nanoemulsion drug delivery system (SNEDDS) by using copaiba oil resin (*C. reticulata*) as a carrier for *t*-DCTN. A stable SNEDDS single-phase nanoemulsion comprising Tween 80 (7%, *w*/*w*) and copaiba oil (0.5%, *w*/*w*) afforded a fine oil-in-water carrier system (SNEDDS-CO). The dropwise solubilization of *t-*DCTN (1 mg) into SNEDDS-CO resulted in the nanoformulation called SNEDDS-CO-DCTN. Transmission electron microscopy (TEM) analysis revealed spherical nanodevices, while particle size, polydispersity index (PDI), and zeta potential measurements indicated small nanodroplets (about 10 nm), uniformly distributed (between 0.1 and 0.2) and negatively charged for both systems. The in vitro kinetic of *t*-DCTN-loaded (SNEDDS-CO-DCTN) analyzed by using simulated conditions of the gastrointestinal microenvironment, as perspective for oral drug delivery, showed a controlled release profile, and corresponded to the Fickian diffusion model. The in vitro antioxidant activity of the samples (*t*-DCTN, SNEDDS-CO, and SNEDDS-CO-DCTN) was confirmed through total antioxidant capacity (TAC), reducing power, copper ion chelation, and hydroxyl radical scavenging assays. The antioxidant activity of SNEDDS-CO-DCTN which contained 1 mg of *t*-DCTN per mL^−1^ of the carrier SNEDDS-CO was similar or even better when compared to the unload *t*-DCTN solubilized in DMSO (10 mg mL^−1^). The SNEDDS formulations herein described were successfully obtained under moderated and controlled conditions, exhibiting effective physicochemical data and release characteristics with huge bioaccessibility for co-loading copaiba oil and *t*-DCTN. The novel colloidal system SNEDDS-CO-DCTN is a potential antioxidant nanoproduct and, from now on, is available for further pharmacological investigations.

## 1. Introduction

**Figure 1:**
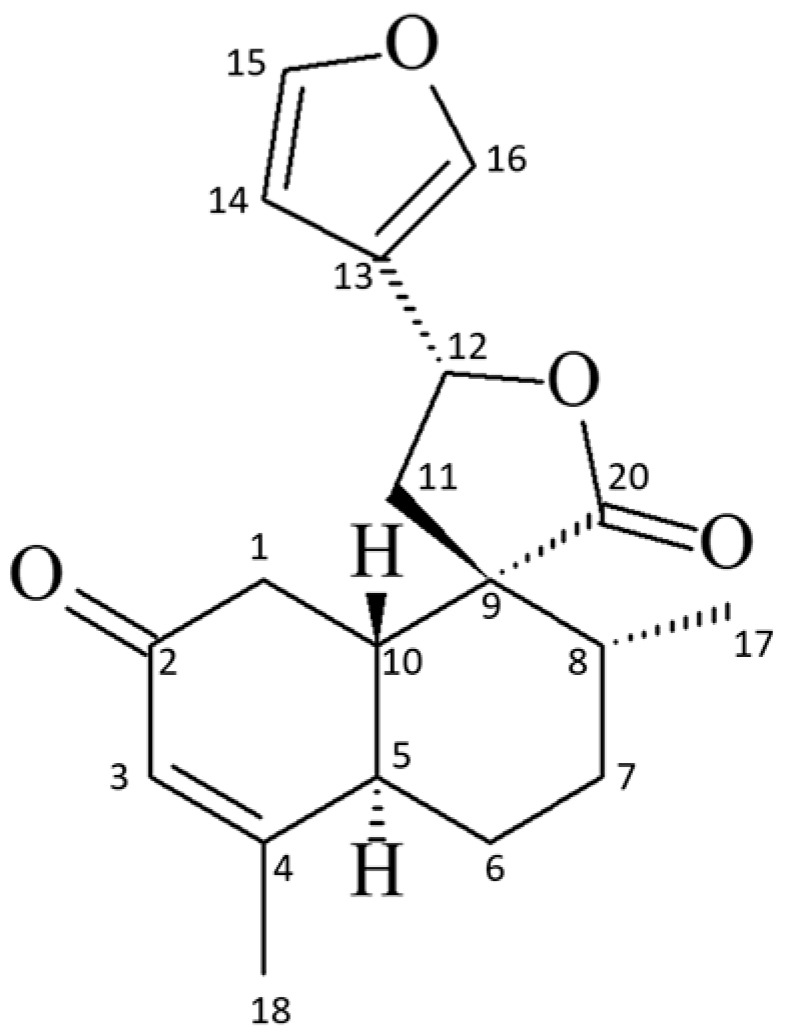
Chemical structure of the 19-*nor*-clerodane *trans*-dehydrocrotonin (*t*-DCTN).

*Croton cajucara* Benth (Euphorbiaceae) is well known from ancient times (about 3000 years) and occurs exclusively in the Amazon region of Brazil, where it is widely used in folk medicine. The strong scientific validations of the medicinal properties of *C. cajucara* enabled this plant to be part of the Brazilian public health program named SUS (Brazil Unified Health System). The stem bark of *C. cajucara* is popularly used as tea or pills to treat several diseases, including diabetes, diarrhea, stomach ache, fever, hepatitis, and malaria [1,2]. The diterpene 19-*nor*-clerodane *trans-*dehydrocrotonin (*t*-DCTN, Figure 1) is the major constituent present in the bark of this *Croton*, and represents one of the most investigated clerodane diterpenoid types in the current literature [3]. This bioactive natural product, along with extracts and fractions of *C. cajucara*, shows a huge variety of pharmacological activities, such as anti-inflammatory [4,5,6], antinociceptive [4,5,6], antiulcerogenic [7], antitumor [8], antiestrogenic [9], hypoglycemic [10,11,12,13], hypolipidemic [10,11,12,13], antigenotoxic [14,15], antimutagenic [16], antitrypanosomal [17,18,19], antileishmanial [17,18], and cardiovascular protective properties [20].

Generally, clerodane diterpenoids are a widespread class of special phytometabolites found in a diversity of plant species from various families and also in organisms from other taxonomic groups. These substances have attracted interest in recent years due to their notable biological activities. The distribution, chemotaxonomic significance, chemical structures, **s**ynthesis for new derivative compounds, and biological activities of clerodane diterpenes have shown potential for their advances in modern biotechnological applications [21,22,23,24,25]. As recently reported by Lima et al. (2024) in a review paper, nanobiotechnological investigations of *C. cajucara* are highlighted in the National Institute of Industrial Property of Brazil (INPI), and among its patents stand out SNEDDS (Self-Nanoemulsion Drug Delivery System) formulations whose oil phase contains the food oil soybean (*Glycine max*), aiming to load *t*-DCTN in low concentration (1 mg–5 mg) for oral pharmacological applications [3]. Since the main pharmacological activity of *C. cajucara* was correlated to *t*-DCTN, the aim of this work was to prepare, characterize, and evaluate the in vivo antioxidant effect of the novel formulation called SNEDDS-CO-DCTN, in which *t*-DCTN (1 mg) was co-encapsulated with the oil resin (1%, *w*/*w*) from *Copaifera reticulata* Ducke. Despite the therapeutic importance of copaiba oil (CO), its bioavailability on a large scale is limited due the low solubility in water and dosage control of this oil extracted from *Copaifera multijuga* Hayne, *Copaifera reticulata* Ducke, *Copaifera langsdorffii* Desf., *Copaifera officinalis* L., *Copaifera paupera* Herzog, and also from unidentified *Copaifera* species [26,27,28,29,30,31,32,33,34]. Additionally, some studies associated copaiba oil with adverse reactions [27,28,29]. In this present work, the antioxidant potential of *t*-DCTN co-encapsulated with copaiba oil resin (SNEDDS-CO-DCTN) was evidenced in the tests of TAC-assay, reducing power, chelating activity of copper ions, and hydroxyl radical scavenging. The aqueous compatibility of these renowned natural products (*t*-DCTN and CO) co-loaded into a SNEDDS system, in lower concentration, was confirmed by physicochemical analysis such as particle size, PDI, and zeta potential, and in vitro kinetic analysis.

### Colloidal Delivery Systems in Improving Drug Bioavailability

Oral drug administration is preferred in conventional therapy of some diseases, being the first form researched for the development of new drugs. However, the lower solubility of drugs in aqueous media and their lack of stability in the first-pass metabolism are the primary limitations in the development of oral medicinal products [35,36,37,38,39,40]. In addition, the pre-systemic metabolism interferes, reducing the drug concentration before it reaches the site of action, demanding elevation of the drug therapeutic dosage. To solve these old problems and create safe bioproducts, novel tools and advanced materials have become available in nanobiotechnology [41,42,43,44]. Indeed, in the last 20 years, nanobiotechnology has been employed in the search for more effective drugs, especially with regard to the assisted delivery (prolonged and controlled) of drugs, and also to allow a decrease in doses, reducing toxicity risks and/or adverse side effects. In this perspective, standing out are colloidal delivery systems such as microemulsion and nanoemulsion, as well as their advanced formulations:—self-microemulsion drug delivery system (SMEDDS) and self-nanoemulsion drug delivery system (SNEDDS)—which increase the drug bioavailability and contribute to the enhancement of drug long-term stability, permeability, and therapeutic functions, affording effectiveness to dissolve and load polar and nonpolar bioactive compounds, maintaining them in a molecular dispersion by controlled and sustained release action [35,36,37,38,41,42,45,46,47,48,49].

The stability of classical colloidal systems (micro- and nano-emulsions) is governed by the physico-chemical properties of components and also the balance of the molecular interactions between the nanodomains of the colloidal system [50,51,52,53,54,55]. The preparation of nanoemulsions requires mechanical energy or energy generated by the chemical potential inherent to the components. High-energy emulsification (dispersion) affords fine nanoemulsions by using mechanical energy through high shear stress, and is generally used in industrial operations through high-pressure homogenizers or ultrasonic generators, which make it possible to control the size and distribution of droplets. Low-energy emulsification (condensation) basically requires the chemical energy stored in the system itself for the formation of emulsions with very small droplets, and goes through the phase transition during the emulsification process, which can occur at constant temperature while varying the composition, or by keeping the composition constant and varying the temperature [45,53,56,57,58,59,60]. Meanwhile, the method of phase inversion temperature (PIT) is based on the change in solubility of nonionic surfactants with the change in temperature [61].

Self-nanoemulsifying colloidal systems can be prepared by using small amounts of non-ionic surfactants (lower than 12%, *w*/*v*); these show (i) different patterns in physical and rheological properties with decreases in droplet size, and suitability for oral ingestion [29,41,42,45,46,47,62]; (ii) resistance to water dilution and pH variation [42,45,47,48]; (iii) less surface tension between the oil and water molecules, raising the formulation stability and easing penetration, due to very fine particle size, favoring the droplet agglomeration, reducing the possibility of creaming or sedimentation [42,46,63]; (iv) ability to effectively solve problems of low drug solubility and oral absorption, and reduce the instability of some colloidal polar systems (O/W) [29,42,45,46,47,62,63]; (v) ability to prevent the high instability of numerous organic substances in the gastrointestinal tract and facilitate intestinal lymphatic transport, conferring advantages over blood absorption such as transport of orally administered drugs through the intestinal lymph, preventing the pre-systemic hepatic metabolism, increasing the drug concentration in the systemic circulation, and increasing the possibility of drug distribution at specific sites to lymphatic organs [41,42,45,46,63].

In this scenario, the novel nanoformulation SNEDDS-CO-DCTN, containing 1 mg of *t*-DCTN per mL^−1^ of the carrier system (SNEDDS-CO), stands out as prospective for oral drug delivery through several pharmacological investigations, and also is available to be largely evaluated on some biological studies, such as cellular culture medium, molecular environment at general organism levels, searching for the drug mechanism of action, aiming at to understanding the interplay between physiological parameters, and the intrinsic properties of the bioactive 19-*nor*-clerodane *trans*-dehydrocrotonin.

## 2. Results and Discussion

The authenticity of copaiba oil resin (*Copaifera reticulata* Ducke) was confirmed by our standardized chromatographic methodology previously reported for some *Copaifera* spices [27,29,64,65,66,67] whose constituents were analyzed as their methyl ester derivative compounds. The identification of the chemical components was carried out by comparing the information obtained in the chromatographic analysis with information available in the Wiley Mass Spectral Databases. Therefore, the analysis was performed by the detection of methyl esters, as derived compounds of the in natura oil resin of *C. reticulata* Ducke (CO). The chromatographic and spectrometric analysis enabled the identification of some sesquiterpene (55.62%) and diterpene (35.47%) constituents analyzed as the CO-methyl-ester derivative compounds. The major identified sesquiterpenes comprise β-bisabolene (20.56%) and α-bergamotene (17.53%), and the minor compounds comprise β-selinene (5.07%), α-selinene (4.34%), β-sesquifelandrene (2.05%), β-farnesene (1.81%), β-caryophyllene (1.23%), and α-yllangene (1.16%). For the diterpene, kaurenoic acid (13.95%), danialic acid (12.42%), cativic acid (6.88%), ent-kaurenol (1.71%), and pinifolic acid (traces) were found.

For copaiba oil studies, although most articles did not detail the investigated species, *C. reticulata* is the most frequently reported [68]. The pharmacological properties of the *Copaifera* genus (Leguminosae-Caesalpinoideae) are related to terpenoid compounds such as sesquiterpenes and diterpenes. In this genus, the main identified sesquiterpenes are β-caryophyllene, caryophyllene oxide, α-copaene, α-humulene, τ-muurolene, β-bisabolene, and β-bisabolol, and the highlighted diterpenes are kaurenol, kaurenoic acid, copalic acid, agathic acid, and hardwiickic acid. It is known that **(i)** β-bisabolene has anti-inflammatory and analgesic proprieties and β-caryophyllene is described as anticancer, anti-inflammatory, and antimicrobial agent [27,65,68,69,70,71,72,73,74,75,76,77,78]; **(ii)** α-bergamotene attracts ectoparasites such as *Melittobia digitata* (wasps), serving as a trapping strategy, warding off pests [79], and acts as a bioactive compound, acts reducing inflammatory cytokines [80]; **(iii)** β-salinene has been found as a major compound in several bioactive herbal oils and shows diversity in both the qualitative and quantitative makeup of some medicinal species which exhibit anti-inflammatory, analgesic, and antipyretic activity [81,82,83]; and **(iv)** kaurenoic acid has been described as a multifunctional substance that exhibits a broad spectrum of biological activities such as diuretic, vasorelaxant, anti-asthmatic, antispasmodic, hypoglycemic, analgesic, anti-inflammatory, anticancer, antimicrobial, and neuroprotective [84,85,86].

### 2.1. Phytochemistry Analysis of Croton cajucara Benth

**Figure 2:**
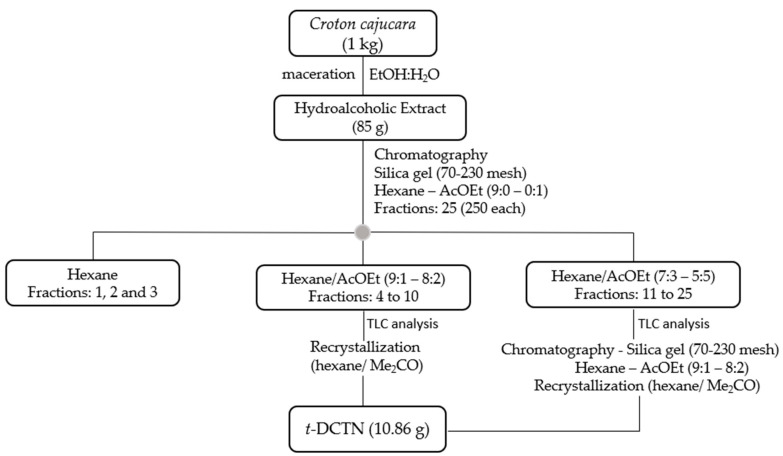
Phytochemical approach for isolation of the *trans*-dehydrocrotonin (*t*-DCTN). EtOH = Ethanol; H_2_O = Water; EtOAc = Ethyl Acetate; Me_2_CO = acetone.

The hydroalcoholic extract (85 g, 8.5%) from the stem bark of *Croton cajucara* (1 kg) was prepared by using maceration procedure with extraction solvent containing a mixture of EtOH:H_2_O (8:2). The classical chromatography method, under a silica gel (70–230 mesh) condition and elution with mixtures of hexane: EtOAc (9:0–0:1), afforded 25 fractions (Figure 2).

The authenticity of the plant material was proven by the isolation and spectroscopic characterization of *t*-DCTN through infrared spectra (IR) and the nuclear magnetic resonance (^1^H NMR) spectrum. As a natural product from herbal species, it was isolated in good yield (10.86 g, 1,17%), as a colorless crystal, and was considered satisfactory by comparison with our previous results [87,88]. The analysis from thin-layer chromatography (TLC), melting point (136–137 °C), and ^1^H NMR indicate that *t*-DCTN showed satisfactory purity.

#### Spectral Data of the 19-*nor*-Clerodane *trans*-Dehydrocrotonin

**IR** ν _max_ cm^−1^ (CHCl_3_): 3120, 2959, 2859, 1748, 1666, 1434, 1504, 1285, 873.(IR spectra available on Supplementary Material).

The infrared (IR) spectra show the presence of a characteristic α,β-unsaturated ketone (1666 cm^−1^) and a furyl moiety (1504 and 873 cm^−1^), and the absorption at 1748 cm^−1^ is related to the presence of a lactone carbonyl group, 1434 cm^−1^ to methylene groups (CH_2_), and 1285 cm^−1^ to C-O.

**^1^H NMR** (CDCl_3_, 600 MHz, δ ppm, *J* Hz): **H1a** δ 2.19 (1H, dd, 15.61, *J* = 13.80); **H1e** δ 2.54 (dd, *J* = 15.61 and 2.80); **H3** δ 5.89 (1H, m, *J* = 1.27 and 1.19); **H5** δ 3.18 (1H, m, *J* = 12.50, 10.70, 3.35, 1.20, and 1.19); **H10** δ1.81 (1H, ddd, *J* = 13.80, 10.70, and 2.80); **H11A** δ 2.42 (1H, dd, *J* = 13.95 and 8.62); **H11B** δ 2.37 (1H, dd, *J* = 13.95 and 8.65); **H12** δ 5.43 (1H, ddd, *J* = 8.65, 8.62, and 0.72); **H14** δ 6.41 (1H, br dd, *J* = 1.83 and 0.89); **H15** δ 7.45 (1H, dd, *J* = 1.83 and 1.66); **H16** δ 7.46 (1H ddd, *J* = 1.66, 0.89, and 0.72); **Me17** δ 1.16 (3H, d, *J* = 6.80); **Me18** δ 1.97 (3H, dd, *J* = 1.27 and 1.20).

The analysis of the ^1^H NMR spectrum (chemical shifts δ in ppm) indicated the presence of a secondary methyl group (Me-17) at δ 1.16 (3H, d, *J* = 6.80 Hz), and a vinyl methyl group at δ 1.97 (3H, *dd*, *J* = 1.27, and 1.20 Hz), confirming the presence of a methyl group attached to sp^2^ carbon (Me-18). The β-substituted furyl group is evidenced at δ 6.41 (1H, br dd, *J* = 1.83, and 0.89 Hz), 7.45 (1H, dd, *J* = 1.83, and 1.66 Hz) and 7.46 (1H, ddd, *J* = 1.66, 0.89, and 0.72 Hz). Hydrogens at position 1 were assigned to be *axial and equatorial*, respectively, δ 2.19 (1H, dd, *J* = 15.61, and 13.80 Hz) and δ 2.54 (1H, dd, *J* = 15.61, and 2.80 Hz), attributed based in their J values. The hydrogen attached to sp^2^ carbon (H3) shows a multiplet at 5.89 (1H, m, *J* = 1.27, and 1.19 Hz). The coupling constant between the hydrogens H5 and H10 (*J* = 10.70 Hz) confirms the *trans*-junction of the decalin system. The coupling constants of the hydrogens H12 and H11 (CH_2_ methylene group) allowed the unambiguous assignments at δ 2.42 (1H, dd, *J* = 13.95, and 8.62 Hz), δ 2.37 (1H, dd, *J* = 13.95, and 8.65 Hz) and δ 5.43 (1H, ddd, *J* = 8.65, 8.62, and 0.72 Hz). ^1^H NMR spectra is available on Supplementary Material. The IR and ^1^H NMR data are consistent with the literature [87,88,89].

Diterpenes have a basic skeleton of 20 carbon atoms and are biogenetically derived from geranyl geranyl pyrophosphate, which results from the head–tail chaining of four isoprene units [90]. Clerodane-type diterpenes originate from the labdanus skeleton by concerted rearrangement, involving consecutive hydride and methyl migrations. Indeed, *trans*-clerodane compounds are obtained by migration of methyl 19 (Me-19) and *cis*-clerodanes by migration of Me-18 [21].

The use of prefixes indicating modifications in the skeleton in relation to the basic structure is quite common, such as *ent*, *seco*, *nor*. The prefix *ent* is used to indicate inversion in all chiral centers, *seco* shows the breakage of some bond in the ring skeleton, and *nor* indicates a missing carbon on the basic skeleton structure. A *nor*-diterpene will have 19 carbon atoms and not 20 as would be expected; bisnor, trinor and tetranor indicate the loss of two, three and four carbon atoms, respectively [21,25]. Natural sources of *nor*-diterpenes exhibit diverse pharmacological activities, including anti-inflammatory, anti-tumor, and antimicrobial effects. Therefore, extensive attention has been drawn toward research on *nor*-diterpenes.

Diterpenes of the clerodane type are widely found in plants belonging to the genus *Teucrium* (Labiatae), followed by the genera *Ajuga*, *Scutellaria*, *Clerodendrum*, and *Croton* [25,91]. The species *Croton schiedeanus* Schlecht, *Croton sonderianus* Müll. Arg., and *Croton cajucara* Benth are the most representative, due to the abundant occurrence of clerodane [24,25,91]. Currently, the 19-*nor*-clerodane *trans*-dehydrocrotonin was correlated with most of the therapeutic indications of *C. cajucara*, and because of the various studies carried out with this compound, it has become the most representative biologically active *nor-*clerodane diterpene [3,91,92,93,94,95,96].

### 2.2. SNEDDS-Copaiba Oil Colloidal Formulation, Characterization, and Stability Analysis

**Figure 3:**
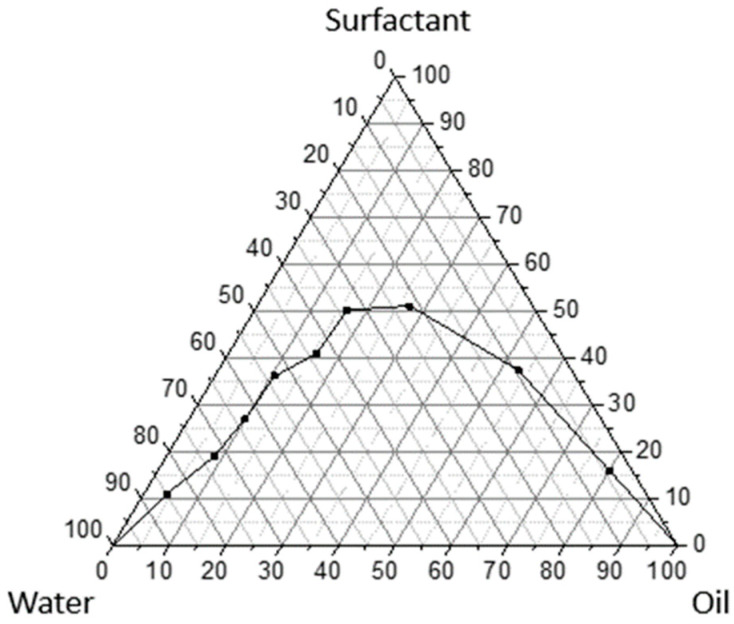
Ternary phase diagram of the SNEDDS-CO system. Surfactant = Tween 80 (7%, *w*/*w*), oil phase (1%, *w*/*w*) containing a mixture of copaiba oil and sunflower oil (1:1), and water (92%, *w*/*w*); SNEDDS-CO = oil-in-water (O/W) colloidal carrier system. The homogeneous and transparent colloidal system is shown in the region above the curvature line.

A stable single-phase SNEDDS system containing copaiba oil (*Copaifera reticulata* Ducke) was produced by using a ternary phase diagram based on determination of the maximum solubility of the active matter (surfactant) in the aqueous and oil phases, by means of mass titrations. The colloidal-based system prepared from the ternary phase diagram (Figure 3) comprising Tween 80 (7%, *w*/*w*) as surfactant, an oil phase (1%, *w*/*w*) containing a mixture of copaiba oil and sunflower oil (1:1), and water (92%, *w*/*w*) afforded a fine oil-in-water (O/W) colloidal system called SNEDDS-CO. Under mild agitation and temperature condition (55 °C to 65 °C), the mixture of surfactant and oil phase was diluted dropwise with double-distilled water, aiming at the formation of a homogeneous and transparent colloidal SNEDDS-carrier system.

**Figure 4:**
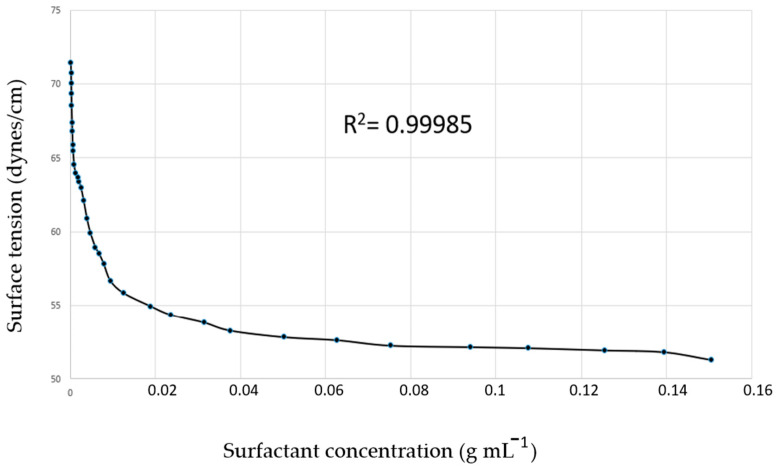
Surface tension of SNEDDS-CO system. SNEDDS-CO = oil-in-water (O/W) colloidal carrier system, containing copaiba oil (0.5%, *w*/*w*).

The surface tension (Figure 4) was 51.29 dynes/cm at 25 °C, showing the cohesion strength between the molecules on the surface of the SNEDDS-CO system. From these data, it was possible to determine the critical micellar concentration (CMC) as 8.315 × 10^−3^ g mL^−1^, which is the surfactant optimal concentration of micelle formation.

Surface forces arise from short-range intermolecular interactions and manifest themselves as longer-range repulsive or attractive forces, and the surface tension, or surface energy is associated with increasing the surface area. Therefore, surface tension is the thermodynamic key property in the interfacial phenomenon resulting from interactions in the region between two different phases (a solid and a liquid, or a gas and a liquid), and also from cohesion forces between the molecules of a given liquid (in this case, resulting from the imbalance of these forces). The asymmetry of the cohesion forces gives rise to the surface energy, which in turn can be evaluated by quantifying the surface tension [97,98,99].

Surfactants, due to their amphiphilic nature, adsorb on interfaces or surface regions in the form of monomers, reducing interfacial tension. This phenomenon is responsible for most of the surfactant properties and structural characteristics of micelles. When small amounts of surfactant are added to water, one part is dissolved as a monomer and another part forms a monolayer at the air–water interface. The molecules of the monolayer remain in equilibrium with the monomers, and each monomer concentration corresponds to a characteristic surface tension. When the concentration of monomers reaches a critical value that determines saturation at the interface, the process of spontaneous formation of molecular aggregates (formation of micelles) is triggered [99,100,101].

The experimental correlation between CMC and the surface properties of surfactants is evaluated, aiming to understand the micellar aggregate formation, which decreases the free energy of the system by reducing the interaction between the hydrophobic groups and the surfactant. Therefore, the CMC provides optimal measurement of surfactant concentration, ensuring the system’s stability. Above the CMC, the concentration of the surfactant results in more micelle formation, but on the other hand, it reduces the free energy of the system. So, below the CMC, the surfactant molecules remain in the form of monomers [102,103,104,105,106,107].

The refractive index of SNEDDS-CO was measured as 1.490, indicating the speed at which light propagates through the system. No deviations were observed in polarized light, which suggests a homogeneous distribution of the components in the formulation. This system was also homogeneous when observed through a light microscope with the phase contrast mode.

**Figure 5:**
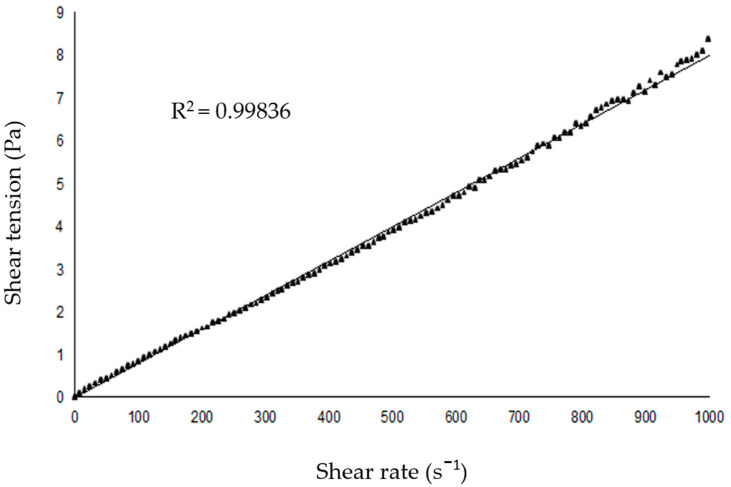
Representative graph of rheological behavior of the SNEDDS-CO system. SNEDDS-CO = oil-in-water (O/W) colloidal carrier system, containing copaiba oil (0.5%, *w*/*w*).

The viscosity of the SNEDDS-CO carrier system was 8 × 10^−3^ cP, which indicates a low resistance to the flow of the emulsion (Figure 5).

#### 2.2.1. Transmission Electron Microscopy Analysis of the SNEDDS-CO System

**Figure 6:**
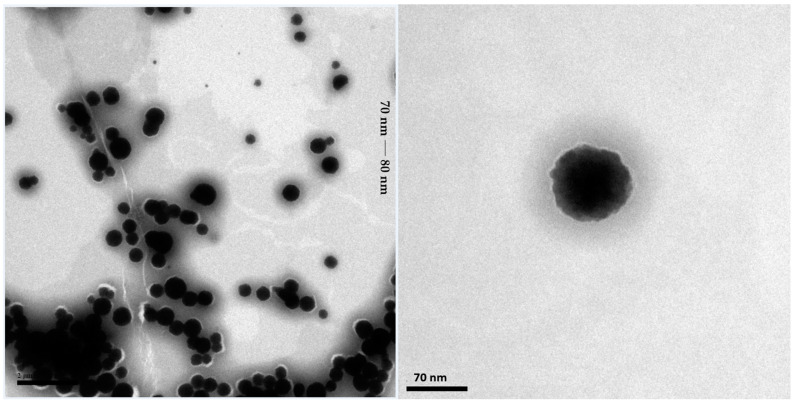
Transmission electron microscopy images of the SNEDDS-CO system. SNEDDS-CO = oil-in-water (O/W) colloidal carrier system, containing copaiba oil (0.5%, *w*/*w*).

The transmission electron microscopy (TEM) analysis of the SNEDDS-CO system, performed aiming to elucidate the morphology and size of micelles, showed the presence of spherical nanoparticles with an average size between 70 nm and 80 nm (Figure 6).

Similar TEM analysis was observed for a SNEDDS system containing coconut oil, Tween 80, and citral as a bioactive, with a higher contrast for the oily cores and a lower contrast for the outer part of the micelle, which could be correlated with the surfactant [108].

Generally, nanoparticles that showed a relative increase in droplet size when compared to the value obtained in the nanoparticle analyzer measurement (dynamic light diffusion/DLS) are justified by the wide difference conditions for TEM analysis, such as non-dilution and the need for drying, that cause modifications [109,110,111].

In the case of the SNEDDS-CO, the mean DLS value was 11.66 nm. Therefore, the increase in TEM analysis was approximately 70%.

In the study developed by Filippov et al. (2023), a detailed analysis was carried out on the discrepancies in the size measurements of nanoparticles obtained by TEM and DLS, identifying percentage deviations that can exceed 200%. The authors associated these differences with factors intrinsic and extrinsic to the samples, such as particle concentration, contrast, surface charge, and morphology [112].

Mondéjar-López et al. (2022) developed chitosan nanoparticles loaded with thymoquinone as preservatives, and observed DLS with sizes ranging between 48.6 nm and 65 nm, while TEM analysis revealed particles with sizes close to 20 nm [113].

The capabilities and limitations of different nanoparticle characterization techniques, including TEM, SEM, AFM, and DLS, were evaluated by Eaton et al. (2017), who observed that the main difference between the methods lies in the distinct environments in which the nanoparticles are analyzed. DLS, for example, measures the polydisperse index and estimates the size of the particles based on the calculation of the hydrodynamic radius, determined by Brownian motion. However, this technique is sensitive to the interaction of particles with other components of the formulation and also to the presence of artifacts or aggregates, which can make it difficult to interpret the results. TEM, on the other hand, provides data dependent on both the type of material and the state of the sample (solid, liquid, dry, or frozen), which makes the direct correlation between the two techniques a huge challenge, since each method reflects specific aspects of the nanoparticles [114].

The physicochemical properties of emulsions depend not only on the component composition of the systems, but also on the methods of their preparation aiming at achieving stable solutions [51,54,115,116].

Since polar colloidal systems are largely used to solubilize hydrophobic substances, using copaiba (CO) oil as the oil phase of a given SNEDDS-CO carrier system could be an optimal choice for its co-encapsulating along with the bioactive terpenoids, such as *trans*-dehydrocrotonin (*t*-DCTN). Indeed, aiming to release this biologic compound contributing to the bioavailability of this 19-*nor*-clerodane diterpene-type into a polar formulation for oral use, the copaiba oil, together with the surfactant phase, facilitated the dissolution of *t-*DCTN, which, in its free form, does not solubilize in aqueous medium. In this sense, the SNEDDS-OC carrier system allows the controlled release of both CO and *t*-DCTN, enabling its lower and prolonged therapeutic action. Specifically, the lower concentration of *t*-DCTN (1 mg) was quantitatively solubilized by mechanical stirring under heating (40 °C to 55 °C) with 1 mL dropwise of SNEDDS-CO. Quantification was performed by spectrophotometer in the UV–visible region using the wavelength (λ_max_ 238 nm). The calibration curve was constructed based on the relationship between concentration versus absorbance with R^2^ = 0.9998, showing that the sample was quantitatively solubilized. Therefore, the loading effectiveness of CO and *t*-DCTN co-encapsulation afforded the nanoproduct called SNEDDS-CO-DCTN (1 mg mL^−1^).

Particle size [dynamic light diffusion/DLS, Ø (nm)], polydispersity index (PDI), and zeta potential (*ζ* mV)) analysis showed, respectively, mean values of 11.66 nm, 0.17, and −3.85 for the carrier system SNEDDS-CO, and 11.29 nm, 0.10, and −3.44 for the nanoproduct SNEDDS-CO-DCTN (Table 1), indicating that there was a small and uniform distribution of the nanodroplets in both systems.

The zeta potential of SNEDDS-CO-DCTN could be a modular small number due the presence of nonionic surfactant (Tween 80) fixed around the oil droplet. So, due the solvation effect at the polar head group of the surfactant and also by the presence of carboxylic acid groups of some acidic molecules from copaiba oil, as well as the carbonyl groups of the chemical structure of *t*-DCTN, it is possible that these SNEDDS systems stabilized through steric rather than electrostatic effects.

The choice of emulsifiers to stabilize emulsions is carried out by using HLB (hydrophilic–lipophilic balance) data, and the effect of surfactant type is determined by the characteristic scale of HLB, as well as the surfactant amount and emulsification methodology [30,31,32,33,34,39,51,117]. Focusing in our previous studies caried out with copaiba oil (*Copaifera langsdorffii* Desf.) loaded into SNEDDS-type formulations composed of Tween 80 (20%), no co-surfactant, and an oil phase (1%) containing copaiba oil mixed (1:1) with coconut oil or sunflower oil, it we found lower data for particle size and zeta potential, such as 5.87 nm and −1.240 (ζ mV) for SNEDDS-CO/coconut oil, and 6.94 nm and −2.460 (ζ mV) for SNEDDS-CO/sunflower oil [29].

In the other hand, the study developed by Nogueira Barradas et al. (2023) addresses the elaboration of a classical nanoemulsion based on *C. officinalis* oil, finding particle diameters ranging from 33.03 ± 1.18 nm to 43.98 ± 4.23 nm, measured by using the DLS method. It was observed that the increase in diameter was intrinsically related to the increase in oil concentration. In order to reduce the discrepancies between the values obtained by TEM and DLS, the authors used the negative staining technique, with phosphotungstic acid as the coloring agent, which helped in the identification of the film created by the surfactant, and promoted an amorphous protection around the particles, favoring their visualization and also preventing collapse and coalescence in the drying stage. So, TEM analyses revealed particles with pseudospherical/oval morphology, with dimensions close to the values obtained by DLS [118].

Venturini et al. (2015) performed particle diameter measurements using the DLS technique, for a formulation based on copaiba oil (unidentified species) co-encapsulated with imiquimod, obtaining, for two proposed formulations, values of 206 ± 6 nm and 177 ± 2 nm. TEM analysis revealed particles with conserved morphology and spherical appearance, being in agreement with the DLS measurements [119].

Carvalho et al. (2022) used a copaiba oil-based nanocarrier system for encapsulation of docetaxel, aiming at to optimize its solubility and bioavailability in anticancer therapy. The DLS particle diameter values were 221.5 ± 2.5 nm (docetaxel-loaded) and 192.6 ± 2.3 nm (docetaxel without encapsulation). TEM analysis of docetaxel-loaded revealed spherical particles with no morphological changes, with dimensions ~200 nm [120].

#### 2.2.2. In Vitro Release Kinetic of trans-Dehydrocrotonin Loaded into the SNEDDS-CO System

**Figure 7:**
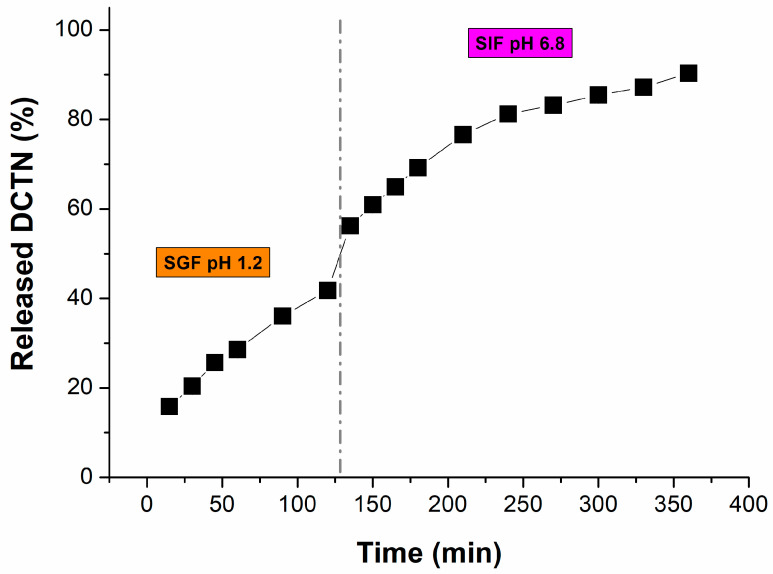
In vitro release profile of *t*-DCTN (percentage released) as a function of time (in minutes) from the SNEDDS-CO-DCTN formulation in the simulated gastric fluid (SGF, pH = 1.2) and simulated intestinal fluid (SIF, pH = 6.8). The drug release diffusion was more favored at the intestinal medium (pH 6.8).

The in vitro release of *trans-*dehydrocrotonin co-encapsulated with copaiba oil (*Copaifera reticulata* Ducke) was determined in two mediums simulating physiological conditions such as **(i)** simulated gastric fluid (SGF, pH = 1.2) during 120 min., and **(ii)** in the simulated intestinal fluid (SIF, pH = 6.8), continued for up to 360 min., by using the dialysis method (Figure 7). This assay is very important to predict the oral bioperformance of the drug delivery system under the human gastrointestinal microenvironment [121].

In the first stage (SGF), the release of *t*-DCTN exhibited a two-phase pattern. The initial phase featured a rapid onset release (*burst* effect) of 25.68 ± 0.02% of *t*-DCTN at 45 min., which can be attributed to the weakly bound or absorbed drug (*t*-DCTN) incorporated in the surface of the carrier system SNEDDS-CO. The second phase is characterized by a more controlled drug release profile, which can be attributed to the diffusion of the *t*-DCTN through the SNEDDS-CO-DCTN formulation, reaching a maximum cumulative release of 41.76 ± 0.01% at 120 min. In the second stage, SIF is released initially at 56.23 ± 0.03% of *t*-DCTN at 135 min., where the sustained profile is maintained until of end of the experiment, with a maximum cumulative release of 90.33 ± 0.01% at 360 min. As evidenced (Figure 7), the process of drug release diffusion was more favored at the intestinal medium (pH 6.8), when compared to the gastric one (pH 1.2), which is possibly associated with *t*-DCTN enhanced solubility at higher values of pH. Phenomena such this present one were observed by Arriagada et al. (2020) and Estime et al. (2010) for other pharmaceuticals [122,123].

**Figure 8:**
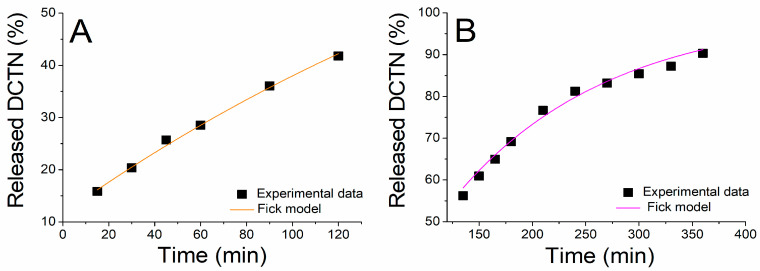
Adjustment of the in vitro release profile of *t*-DCTN from the SNEDDS-CO-DCTN formulation (squares) to Fickian’s kinetic model (lines): (**A**) (SGF, simulated gastric fluid) and (**B**) (SIF, simulated intestinal fluid).

The release kinetic data were adjusted by the non-linear regression by using some mathematical models, but the equations from the Fickian kinetic model provided the best correlation coefficient (R^2^ = 0.98), as shown in Figure 8. This result indicated that the diffusion was considered the main release mechanism. The kinetic parameters calculated from the Fickian model were k = 0.00355 ± 0.00007 min^−1^ for SGF, and k = 0.00694 ± 0.00036 min^−1^ for SIF.

In our previous work, the release of *trans*-dehydrocrotonin loaded at the highest dose (10 mg) into a vesicular delivery system (liposome formulation) also showed a controlled drug profile by diffusion, in agreement with the Fickian kinetic model. In this case, the *t*-DCTN nanoformulation employed a simulated condition for parenteral application (7.4 phosphate buffer). Indeed, there was improvement in *t*-DCTN antitumor activity against Sarcoma 180, and also a reduction in the drug hepatotoxicity risk in Swiss mice. Thus was evidenced an important advance for enabling *t*-DCTN nanoformulations to be used in the nanotherapy of cancer [124,125].

In this present work, *trans*-dehydrocrotonin loaded into the colloidal SNEDDS-CO carrier system (1 mg mL^−1^) was bioaccessible to perform, for the first time, simulated conditions for its oral delivery. So, aiming at a preliminary application of the formulation, SNEDDS-CO-DCTN effectiveness was evaluated on in vitro antioxidant assays.

Despite the SNEDDS drug delivery system’s advantages, and hundreds of in vivo studies carried out in several animal models, whose results showed the improvement of the drugs’ solubility, and the SNEDDS formulation’s being able to bypass lymphatic transport with promising results, this type of colloidal system faces limitations in the sense that there are not many pharmacokinetic studies carried out in humans, using SNEDDS-type systems administered orally. Therefore, the results of this present work are promising for oral *t*-DCTN load into a stable SNEDDS formulation.

### 2.3. In Vitro Antioxidant Analysis of trans-Dehydrocrotonin Loaded into the SNEDDS-CO System

#### 2.3.1. Determination of Total Antioxidant Capacity

**Figure 9:**
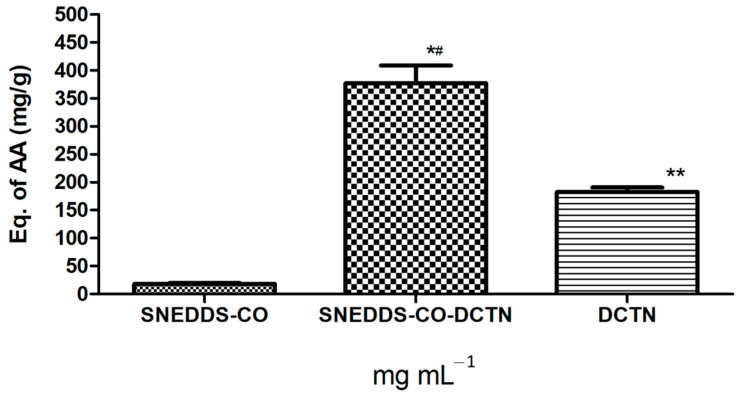
Determination of total antioxidant capacity equivalent to ascorbic acid (AA) mg/g of the SNEDDS systems and the unload *t-*DCTN. * and ** mean the statistical difference between the samples related to SNEDDS-CO system (*p* < 0.001). # means the statistical difference between SNEDDS-CO-DCTN system and *t*-DCTN-DMSO solution (*p* < 0.001). SNEDDS-CO = oil-in-water (O/W) colloidal carrier system, containing copaiba oil (0.5%, *w*/*w*). SNEDDS-CO-DCTN containing 1 mg of *t*-DCTN per mL of the carrier SNEDDS-CO. DCTN = solution containing 10 mg of *t*-DCTN per mL of DMSO.

In this assay, SNEDDS-CO-DCTN formulation (*t*-DCTN, 1 mg mL^−1^) and the unload *t*-DCTN (10 mg) solubilized in DMSO, showed TAC activity strongly higher than the result observed for the SNEDDS-CO carrier system (Figure 9).

In this assay, the ascorbic acid has the ability to transfer electrons and bind to metal ions, providing protection against oxidation in aqueous media within cells. Indeed, when antioxidant defenses are weakened, the body cells and tissues become more prone to develop dysfunction and/or disease [126]. Then, the maintenance of adequate antioxidant levels is essential to manage or even prevent a great number of disease conditions. In this sense, the measurement of TAC (total antioxidant capacity) of biological samples is a useful tool for investigating biological fluids and monitoring several diseases associated with oxidative stress, such as type 2 diabetes [127], chronic hepatitis C [128,129], cardiovascular and inflammatory bowel diseases [130]. This result reinforces the therapeutic potential of *t*-DCTN previously evaluated in in vivo models for hypoglycemic and hypolipidemic activities [10,11,12,13], and also its cardiovascular protective property [20].

#### 2.3.2. Antioxidant Capacity via Reducing Power

**Figure 10:**
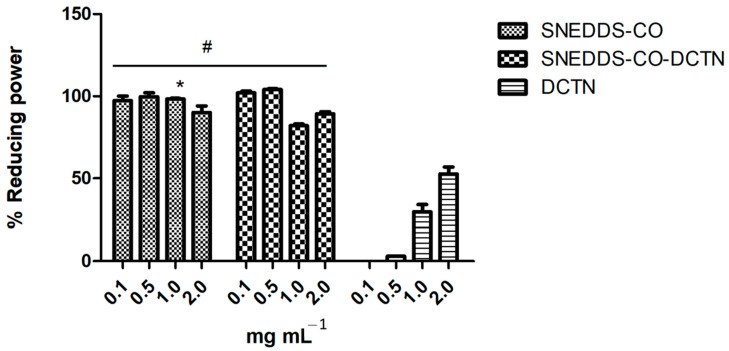
Antioxidant capacity via reducing power of SNEDDS systems and the unload *t-*DCTN. * means the statistical difference between SNEDDS-CO and SNEDDS-CO-DCTN systems at 1.0 mg mL^−1^ (*p* < 0.001). # means the statistical difference of both SNEDDS-CO and SNEDDS-CO-DCTN at the same concentrations by comparing with *t*-DCTN-DMSO solution (*p* < 0.001). SNEDDS-CO = oil-in-water (O/W) colloidal carrier system, containing copaiba oil (0.5%, *w*/*w*). SNEDDS-CO-DCTN containing 1 mg of *t*-DCTN per mL of the carrier SNEDDS-CO. DCTN = solution containing 10 mg of *t*-DCTN per mL of DMSO.

In this assay, the samples (SNEDDS-CO, SNEDDS-CO-DCTN, and *t*-DCTN-DMSO solution) were evaluated at different concentrations (0.1, 0.5, 1.0, and 2.0 mg mL^−1^). For the SNEDDS-CO sample, the reduced power is over 94%. Specifically, 100% reducing activity was observed at the 0.5 mg mL^−1^, 98.5% at 1.0 mg mL^−1^, 97.33% at 0.1 mg mL^−1^, and 94.0% at 2.0 mg mL^−1^ (Figure 10). For the SNEDDS-CO-DCTN sample, the reducing power was observed above 80%—specifically, 99.33% at the concentration of 0.1 mg mL^−1^, 99.66% at 0.5 mg mL^−1^, 82% at 1 mg mL^−1^, and 89.33% at 2 mg mL^−1^ (Figure 10). These results were compared with *trans*-dehydrocrotonin solubilized in DMSO (10 mg mL^−1^), and it was possible to evidence the bioavailability improvement of *t*-DCTN loaded into the SNEDDS-CO system (Figure 10).

Many antioxidants are proven to undergo an electron-transfer mechanism upon exerting their antioxidant functions. Therefore, reducing power is a measure of a substance’s ability to transfer electrons in a neutral condition [131,132]. In this test, the SNEDDS-CO system showed greater capacity to donate electrons at all concentrations with no statistical difference compared with the product SNEDDS-CO-DCTN at 0.1 mg mL^−1^ (99.33%) and 0.5 mg mL^−1^ (99.66%). These data indicate that the SNEDDS formulations showed high reducing activity, which can be associated with an antioxidant property via free radical neutralization.

#### 2.3.3. Copper Ion Chelation Method

**Figure 11:**
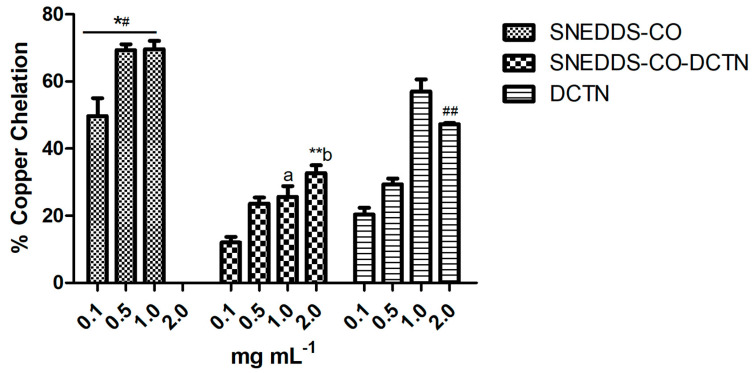
Antioxidant capacity via copper ion chelation of SNEDDS systems and the unload *t-*DCTN. * means the statistical difference between SNEDDS-CO and SNEDDS-CO-DCTN systems, at 0.1 to 1.0 mg mL^−1^ (*p* < 0.001). ** means the statistical difference between SNEDDS-CO-DCTN and SNEDDS-CO systems, at 2 mg mL^−1^ (*p* < 0.001). # means the statistical difference between SNEDDS-CO system and *t*-DCTN-DMSO solution, at 0.1 to 1.0 mg mL^−1^ (*p* < 0.001). ## means the statistical difference between *t*-DCTN-DMSO solution and SNEDDS-CO system, at 2 mg mL^−1^ (*p* < 0.001). a, b mean the statistical difference between SNEDDS-CO-DCTN and *t*-DCTN-DMSO solution, at 1 mg mL^−1^ and 2 mg mL^−1^, respectively (*p* < 0.001). SNEDDS-CO = oil-in-water (O/W) colloidal carrier system, containing copaiba oil (0.5%, *w*/*w*). SNEDDS-CO-DCTN containing 1 mg of *t*-DCTN per mL of the carrier SNEDDS-CO. DCTN = solution containing 10 mg of *t*-DCTN per mL of DMSO.

In this assay, the SNEDDS-CO-DCTN showed low antioxidant activity at all tested concentrations: 8.66% (0.1 mg mL^−1^), 23.66% (0.5 mg mL^−1^), 25.66% (1 mg mL^−1^), and 31.50% (2 mg mL^−1^). Comparatively, SNEDDS-CO at 0.1 mg mL^−1^ (49.66%), 0.5 mg mL^−1^ (69.33%), and 1 mg mL^−1^ (69.33%) showed higher potential activity (Figure 11). On the other hand, the *t*-DCTN-DMSO solution showed great result, at 1 mg mL^−1^.

The induction of oxidative stress is an imbalance between the production of radical species and the antioxidant defense systems, causing oxidative damage to proteins, lipids, and DNA [133]. In this sense, ligand antioxidant properties complexed by selected metals may significantly affect the free radical neutralization [134,135]. Considering the results of the carrier system at 1 mg mL^−1^ and also the *t*-DCTN-DMSO solution at 1 mg mL^−1^ (Figure 10), these samples also could be extensively applied on in vivo models associated with oxidative stress such as type 2 diabetes [127] and chronic hepatitis [129,130].

#### 2.3.4. Hydroxyl Radical Sequestration Capability

**Figure 12:**
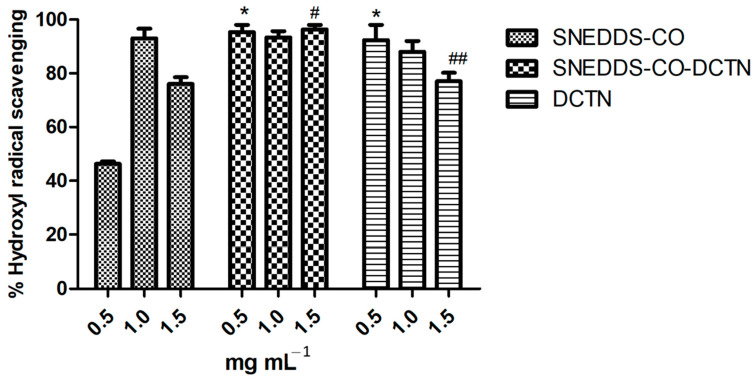
Hydroxyl radical sequestration capability of SNEDDS systems and the unload *t-*DCTN. * means the statistical difference of SNEDDS-CO-DCTN system and *t*-DCTN-DMSO solution by comparing with SNEDDS-CO system, at 0.5 mg mL^−1^ (*p* < 0.001). # means the statistical difference between SNEDDS-CO-DCTN and SNEDDS-CO systems, at 1.5 mg mL^−1^ (*p* < 0.001). ## means the statistical difference between SNEDDS-CO-DCTN and *t*-DCTN-DMSO solution, at 1.5 mg mL^−1^ (*p* < 0.01). SNEDDS-CO = oil-in-water (O/W) colloidal carrier system, containing copaiba oil (0.5%, *w*/*w*). SNEDDS-CO-DCTN containing 1 mg of *t*-DCTN per mL of the carrier SNEDDS-CO. DCTN = solution containing 10 mg of *t*-DCTN per mL of DMSO.

In this test, the SNEDDS-CO system at concentrations 1.0 mg mL^−1^ and 1.5 mg mL^−1^ showed 94% and 76% of antioxidant capacity, respectively (Figure 12). On the other hand, the highest results were noted for SNEDDS-CO-DCTN and *t*-DCTN-DMSO solution at all tested concentrations (0.5, 1.0, and 1.5 mg mL^−1^).

It is important to highlight that copaiba oil (0.5%) from *Copaifera langsdorffii* Desf., mixed with coconut oil (0.5%) or with sunflower oil (0.5%), was previously loaded into SNEDDS systems and then assayed for its antioxidant property. The reducing power and hydroxyl radical (^•^OH)-formation inhibition previously observed for the SNEDDS-CO/coconut oil system, were, respectively, 46.48% and 84.11%, and for the SNEDDS-CO/sunflower system, were, respectively, 52.46% and 74.48% [29].

Since ^•^OH causes oxidative DNA damage that compromises the integrity and function of cell membranes and reacts with almost all organic biomolecules found in living organisms [131,132], certainly, these SNEDDS systems based on copaiba oil from *Copaifera langsdorffii* Desf. and *Copaifera reticulata* Ducke become important targets to combat cell lipoperoxidation.

## 3. Materials and Methods

The herbal resource copaiba oil (*Copaifera reticulata* Ducke), the stem bark of *Croton cajucara* Benth (Euphorbiaceae), and the sunflower oil were commercially obtained. Copaiba oil and *C. cajucara* samples were purchased in traditional markets specialized in natural products located in the Amazon region of Brazil, respectively at Manaus and Belém cities. Complying with the requirements of the New Brazilian Biodiversity Law (Law 13.123/2015), this work was registered in the National Management System of Genetic Heritage and Associated Traditional Knowledge (SISGEN) under number AFF8812.

The solvents acetone (Me_2_CO), chloroform (CHCl_3_), ethanol (EtOH), ethyl acetate (AcOEt), hexane, and methanol (MeOH), all of analytical grade, as well as all other used chemicals, such as ammonium molybdate, ascorbic acid, copper II sulfate pentahydrate, ethylene-di-amine-tetra-acetic acid (EDTA), ferric chloride, ferrous sulfate heptahydrate (FeSO_4_·7H_2_O), ferrous sulphate II, gallic acid, nitroblue tetrazolium (NBT), phosphate buffer, potassium ferricyanide, pyrocatechol violet, riboflavin, sodium phosphate, sodium salicylate, sulfuric acid, trichloroacetic acid, and Tween 80, were purchased from Sigma Aldrich Inc. (St. Louis, MO, USA) or Merck (Darmstadt, Germany).

The melting points were determined with a Kofler (Jasco DIP-370) apparatus, and they have not been corrected. IR spectra (CHCl_3_) were found with a Perkin–Elmer FT-16PC spectrophotometer, and UV spectra (MeOH) with GBC UV/VIS 911A CG or Thermo Electron Corporation/Nicolet Evolution 100) instruments (London, UK and Boston, MA, USA). The NMR ^1^H and all 2D experiments were recorded on a Bruker–Avance spectrometer (600 MHz for ^1^H) (Bruker, Millerica, MA, USA). The deuterated solvent (CDCl_3_) used in NMR spectroscopy was from Merck brands.

### 3.1. Chromatography Analysis of Copaifera reticulata Ducke

Characterization of copaiba oil (CO) after its chemical esterification was performed using gas chromatography high resolution analyses with flame ionization detection (GC-FID) and coupled with mass spectrometry: Trace CG Ultra, Thermo Scientific (Eindhoven, The Netherlands) and mass spectrometry detector (DSQ II, Thermo Scientific) (Eindhoven, The Netherlands) with quadrupolanalyser and auto-injetor (AI 3000, Thermo Scientific) (Eindhoven, The Netherlands). Mass spectra were obtained by electron impact (70 eV), from 40 to 400 u.m.a. The CO sample (5.0 mg) was derivatized in situ using trimethylsilildiazomethane (TMSD), converting the diterpene carboxylic acids into the corresponding methyl esthers. Split injections (1:20) were performed in DB-1 dimethylpolisiloxane (25 m × 0.25 mm × 0.25 μm) and zebron ZB-5ms (Phenomenex-20 m × 0.18 mm × 0.18 μm) columns, using He as carrier gas at 2 mL min^−1^. Oven temperature was programmed from 120 °C to 150 °C at 3 °C.min^−1^, followed by another heating ramp until 290 °C, at 15 °C min^−1^. Detector and injector temperatures were set at 300 °C and 270 °C, respectively. Two standard mixtures were injected at this same condition: a homologous series of linear hydrocarbons from tridecane to heptadecane (C13 to C17), and a mixture containing the sesquiterpenes caryophyllene, humulene, and caryophyllene oxide. The homologous series of hydrocarbons were applied to obtain the Linear Retention Index (LRI) of the copaiba oil constituents, and then compared with the literature. The mixture of these three sesquiterpenes, very common constituents from copaiba oils, was used to correct the LRI obtained and compare them with literature data. Mass spectrometry (MS) experiments were useful to confirm the identification of the sesquiterpenes by comparing their mass spectra with an automatic database (NIST) and also to obtain the diterpene methyl esther mass spectra and compare all of them with mass spectra data from previously isolated substances from copaiba oils that were stored, and to compose a personal data library. The applied methodology is accordance with our previous reported studies [29,133,134].

### 3.2. Chromatography Analysis of Croton cajucara Benth

Stem bark (1 kg) of *C. cajucara* was dried at 37 °C in an oven with controlled temperature and constant renewal of air for four days, and its coarse milling was performed using an SM 300 cutting mill (Grupo Imetal, Santa Catarina, Brazil) operating at 3000 rpm and equipped with a 2 mm grid. The pulverized plant was kept in an air-tight container until before being used. The maceration extraction ratio of 1:10 (*w*/*v*) was applied to prepare the hydroalcoholic extract (EtOH/distilled water).

Classical column chromatography was performed with silica gel (70–230 mesh) as adsorbent, and Thin Layer Chromatography (TLC) was carried out using silica gel 60 H and, revelation was employed with sulfuric acid/methanol (1:1) reagent. TLC was carried out on 0.25 mm layers of silica gel PF 254 (Merck). TLC also was revealed by UV-Vis radiation at wavelength of 254 and 356 nm. The hydroalcoholic EtOH/H_2_O (8:2) extract, after solvent reduction (85 g, 8.5%), was submitted to a chromatographic procedure and afforded 25 fractions eluted with hexane or mixtures of hexane/EtOAc at different ratios of increasing polarity (Figure 2).

The non-polar fractions (F1, F2, and F3) were eluted with hexane, and fractions F4 to F25 were eluted with mixtures of hexane/EtOAc (9:1 to 5:5), and then submitted to recrystallization or submitted to another chromatographic fractionation and then recrystallization, affording 10.86 g (0.17%) of *trans*-dehydrocrotonin (*t*-DCTN). For the TLC analysis mobile phase, mixtures of hexane/EtOAc t (8:2, 7:3, and 6:4) were applied, and revelation was employed with sulfuric acid/methanol (1:1) reagent. Additionally, TLC was also revealed using UV-Vis radiation at wavelength of 254 nm and 356 nm.

The structural elucidation of *t*-DCTN was analyzed by spectroscopic method in the infrared (IR) region, and also proton nuclear magnetic resonance spectra (^1^H NMR).

### 3.3. SNEDDS-Copaiba Oil Colloidal Formulation

The ternary phase diagram was constructed using the surfactant mass titration methodology into the aqueous and oily phases in order to obtain a polar O/W nanoemulsion region. The phase diagram was prepared with Tween 80 as a surfactant, copaiba oil (*Copaifera reticulata* Ducke) and sunflower oil (*Helianthus annuus*) as the oil phase, and doublydistilled water. The surfactant was mixed with the oil phase (weight ratios) respectively: 9:1, 8:2, 7:3, 6:4, 5:5, 4:6, 3:7, 2:8, and 1:9. All mixtures were diluted dropwise with distilled water (*w*/*v*), and the nanoemulsion region of the copaiba oil (CO) colloidal system was produced by using the mechanical stirring Vortex (IKA, Staufen, Germany) at ambient temperature.

The colloidal-based system was composed of 7% surfactant (Tween 80), 1% vegetable oil (a mixture of copaiba oil and sunflower oil, at a 1:1 ratio), and 92% doubly-distilled water, under heating (55 °C to 65 °C). After the physicochemical characterizations by using polarized light microscopy, pH, conductivity, refractive index, droplet size, rheological behavior, and surface tension analysis, the formulation called SNEDDS-CO containing 1% of oil phase (0.5% of CO and 0.5% of sunflower oil) was characterized as SNEDDS-type system.

The pH was measured by using a pre-calibrated pH meter PG-2000 (Gehaka, São Paulo, SP, Brazil) at 25 °C. The electrical conductivity of the samples was measured using a DM-32 conductivity meter (Digicrom Analytical, Campo Grande, SP, Brazil) with a cell constant of 0.11 cm^−1^. The measurements were performed at 25 °C.

The refractive index of the carrier SNEDDS-CO system was determined using Abbe’s refractometer (Bellingham plus Stanley Limited, England) at 25 °C, and the rheological property was determined using an oscillatory Haake Mars rheometer (Thermo Fisher Scientific, Karlsruhe, Germany, cup Z43 DIN 53018 and rotor Z41 DIN 53018). The temperature was kept at 25 °C using a thermostatic bath. Analyses were carried out by applying a shear rate sweep from 0 to 10^3^ s^−1^.

The surface tension of SNEDDS-CO was carried out using a SensaDyne tensiometer (model QC-6000, Milwaukee, WI, USA) employing the maximum pressure bubble technique, using nitrogen as gas phase. The results of the surface tension assay, expressed in mN m^−1^ (or dynes cm^−1^), were analyzed with SensaDyne tensiometer software, version 1.21.

The transmission electron microscopy (TEM) analysis was performed with high-resolution JEM-1230 JEOL equipment to identify nanoparticle images. The analysis was carried out with an acceleration voltage of 300 kV and a magnification range of 50 to 800 thousand times. In order to observe the morphology of the SNEDDS-CO carrier system, a drop of this solution was introduced into a 200-mesh copper grid, and then a drop of 5% phosphotungistic acid was added to this same grid as a contrast agent. And then, it was left to rest for 4 h, and then, the grid was analyzed via TEM. The descriptive methodology for particle size, polydispersity index, and zeta potential analysis for both SNEDDS-CO and the nanoproduct SNEDDS-CO-DCTN are described below (4.5).

### 3.4. Loading of trans-Dehydrocrotonin and Effectiveness of the Solubility in the SNEDDS-CO System

The lower concentration of *t*-DCTN (1 mg) was solubilized dropwise with 1 mL of SNEDDS-CO by mechanical stirring under heating (40 °C to 55 °C), affording the nanoproduct called SNEDDS-CO-DCTN (1 mg mL^−1^). The loading effectiveness of *t*-DCTN was evaluated by the minimum and maximum solubility method. Quantification was performed by spectrophotometer (Thermo Electron Corporation/Nicolet Evolution 100) (London, UK) in the UV–visible region using wavelength (λ) at 200 to 400 nm for determining the λ_max_ (observed at 238 nm). To obtain the calibration curve, the correlation of *t*-DCTN concentration versus absorbance was analyzed using four samples. To determine the minimum and maximum solubility of *t*-DCTN in the SNEDDS-CO carrier system, two masses (1 mg mL^−1^ and 5 mg mL^−1^) were used, and mechanical stirring under heating (40 °C to 55 °C) was applied. After cooling at room temperature, the samples were vortexed for 10 min, followed by stirring in ultrasound for 10 min at room temperature. After equilibration, samples were filtered, and diluted as required with distilled water. Then, the solutions were analyzed in the UV-Vis spectrophotometer at a wavelength of 238 nm.

### 3.5. Particle Size, Polydispersity Index, and Zeta Potential Analysis

The physicochemical characterizations of the nanoproduct SNEDDS-CO-DCTN were conducted through analysis of particle size (Ø), polydispersity index (PDI), and zeta potential (*ζ*), following the methodology previously [124,125], and the obtained data were compared with those observed for SNEDDS-CO.

Micelle dispersion was measured using photon correlation spectroscopy with a Zetasizer Nano-ZS90 (Malvern, Worcestershire, UK). For Ø and PDI analyses, 100 µL of the formulations were diluted in 900 µL of purified water. Measurements were performed at 25 °C with a fixed 90° scattering angle, and results were reported as the mean hydrodynamic diameter of the micelles (nm). Zeta potential (*ζ*) was measured after diluting 50 µL of the formulations in 950 µL of purified water. Surface charge (mV) was evaluated using the Zetasizer Nano-ZS90 (Malvern, Worcestershire, UK).

### 3.6. In Vitro Release Kinetics

The in vitro release kinetics of *t*-DCTN loaded into the nanoproduct SNEDDS-CO-DCTN were analyzed using the dialysis technique under sink conditions in simulated gastric fluid (SGF, pH = 1.2) and in simulated intestinal fluid (SIF, pH = 6.8) at 37 °C. An aliquot (1 mL) of the formulation was withdrawn and placed in a dialysis membrane (Viskase Lombard, USA), which were sealed and suspended in the simulated buffered medium (100 mL). The system was maintained under stirring (100 rpm) and heating (37 °C). Aliquots of 1 mL of the release fluid were collected (replaced by 1 mL of fresh buffer) at predetermined times over 120 min for SGF and continued for up to 360 min for SIF. The amount of *t*-DCTN released as a function of time was determined spectrophotometrically at 238 nm using a standard curve in concentrations ranging from 1 to 20 µg mL^−1^. *t*-DCTN release kinetics were performed in triplicate, and the values were expressed as mean ± standard deviation for the concentration in percentage of cumulative drug released.

The release profile was evaluated by non-linear regression for the mathematical correlation to Fickian’s law, uni-exponential using the following equation:

M_t_/M_∞_ = 1 − be^−kt^

where M_t_ and M_∞_ are the amounts of drug released at a given time t, and the total drug released from SNEDDS-CO-DCTN system, respectively; k is the rate constant associated with the diffusion coefficient of the drug loaded [124,125].

### 3.7. Antioxidant Activity Assays

To evaluate the antioxidant activities, in vitro antioxidant assays applied to the SNEDDS formulations (SNEDDS-CO and SNEDDS-CO-DCTN) and *t*-DCTN solubilized in DMSO (10 mg mL^−1^) were performed by determination of total antioxidant capacity (TAC), reducing power, copper ion chelation, and hydroxyl radical scavenging, according to the methodologies previously described [135,136,137]. The tests were performed in triplicate.

#### 3.7.1. Determination of Total Antioxidant Capacity (TAC)

Briefly, the solution (1 mL) containing the samples, ammonium molybdate (4 mM), sodium phosphate (28 mM), and sulfuric acid (0.6 M) were added into a tube, stirred, and incubated (100 °C, 90 min.). The tubes were cooled and were read at the 695 nm wavelength. The standard used was ascorbic acid (AA) and the results were expressed as AA equivalent per gram of sample [135,136].

#### 3.7.2. Reducing Power

Briefly, phosphate buffer (0.2 M, pH 6.6) containing potassium ferricyanide (1%) was mixed with samples in different concentrations (0.05 to 2 mg mL^−1^) to a final volume of 1.0 mL. After 20 min., at 50 °C, the reaction was stopped with 10% trichloroacetic acid (TCA). One milliliter of ferric chloride (0.1% *w*/*v*) in distilled water was added to the mixture, and the absorbance was measured at 700 nm [135]. The results were expressed as percentage activity of 0.1 mg/mL ascorbic acid (standard), which corresponded to 100% activity.

#### 3.7.3. Copper Chelation

The test was performed in 96-well microplates with a reaction mixture containing different concentrations of samples (0.1 to 2 mg mL^−1^), copper II sulfate pentahydrate (50 mg mL^−1^), and pyrocatechol violet (4 mM). All wells were homogenized with the aid of a micropipette, and the solution absorbance was measured at 632 nm [137]. The ability of the samples in chelating the copper ion was calculated using the following equation:

Chelating effect (%) = (Absorbance of blank) − (Absorbance of the sample)/(Absorbance of the blank) × 100

#### 3.7.4. Hydroxyl Radical Scavenging Assay

The OH radical scavenging activity of samples was investigated using Fenton’s reaction (Fe^2 +^ + H_2_O_2_ - > Fe^3 +^ + OH^−^ + OH). The data were expressed as the inhibition rate. For OH production, the samples (at different concentrations) were added to 3 mL sodium phosphate buffer (150 mM, pH 7.4), which contained 10 mM FeSO_4_.7H_2_O (ferrous sulfate heptahydrate), 10 mM EDTA 2 mM sodium salicylate, and 30% H_2_O_2_. In the control, sodium phosphate buffer replaced H_2_O_2_. After treatment of 37 °C for 1 h, OH radicals were detected by monitoring absorbance at 510 nm using a microplate reader. Gallic acid was used as a positive control [136].

## 4. Conclusions

The authenticity of copaiba oil resin (*Copaifera reticulata* Ducke) was confirmed by GC-MS and standards, identifying sesquiterpenes (55.62%) and diterpene (35.47%) as the major components. Key sesquiterpenes included β-bisabolene and α-bergamotene, while diterpenic acids such as kaurenoic, danielic, cativic, and pinifolic acids were also detected. The diterpene 19-nor-clerodane *trans*-dehydrocrotonin (t-DCTN) was isolated from *Croton cajucara* and fully characterized through FTIR and ¹H NMR spectroscopy.

A stable single-phase colloidal system was developed based on a ternary phase diagram consisting of Tween 80 as a surfactant, an oil phase comprising a 1:1 mixture of copaiba oil and sunflower oil, and water (92%). This formulation produced a fine oil-in-water nanoemulsion designated as the SNEDDS-CO carrier system. The bioactive *t*-DCTN (1 mg) was loaded in the carrier SNEDDS-CO under mechanical stirring and heating, yielding the nanoproduct SNEDDS-CO-DCTN (1 mg mL^−1^). The physicochemical properties and in vitro kinetic profiles of SNEDDS-CO-DCTN were thoroughly characterized, demonstrating sustained *t*-DCTN release, with a maximum cumulative release of 90.33 ± 0.01% over 360 min, simulating conditions for oral delivery system.

Both the SNEDDS-CO and SNEDDS-CO-DCTN formulations exhibited significant antioxidant activity across all in vitro assays. SNEDDS-CO-DCTN showed superior results in total antioxidant capacity (TAC), reducing power, and hydroxyl radical scavenging activity. Meanwhile, SNEDDS-CO demonstrated greater efficacy in copper ion chelating activity.

These findings highlight the effective bioaccessibility of *t*-DCTN co-loaded with copaiba oil resin (*Copaifera reticulata* Ducke) and *t*-DCTN into the SNEDDS-based system and are available for further in vitro and in vivo models for cancer nanotherapy investigations.

## 5. Patent

De Oliveira Netto, J. R.; Corrêa, N. P.; De Araujo, L.B.A.; Paiva, W. De S.; Rocha, H. A. De O.; Morais LIMA, W. De A.; Daniel Macedo, C. Dos S.; Santos Magalhães N. S.; Do Nascimento, J. H. O.; Da Veiga Junior, V. F.; Maciel, M. A. M. Formulação Antioxidante com Potencial Imunomodulador contendo Bioativos de *Copaifera reticulata* Ducke (Óleo Resina de Copaíba) e *Croton cajucara* Benth (*trans*-Desidrocrotonina). 10 April 2024. Brazil, INPI Patent BR102024020730-0 (Instituto Nacional de Propriedade Intelectual).

## Figures and Tables

**Table 1:** Particle size, PDI, and zeta potential analysis of the SNEDDS systems.

	Triplicate Analysis	Ø (nm)	PDI	*ζ* (mV)
	A	11.15	0.083	−3.60
SNEDDS-CO-DCTN	B	11.31	0.107	−3.63
	C	11.43	0.130	−3.11
	D	11.46	0.135	−4.13
SNEDDS-CO	E	11.71	0.188	−3.39
	F	11.81	0.200	−4.04

Ø (nm) = particle size (dynamic light diffusion/DLS); PDI = polydispersity index; *ζ* = zeta potential; SNEDDS-CO = oil-in-water (O/W) colloidal carrier system, containing copaiba oil (0.5%, *w*/*w*); SNEDDS-CO-DCTN containing 1 mg of *t*-DCTN per mL of SNEDDS-CO.

## Data Availability

No new data were created or analyzed in this study. Data sharing is not applicable to this article.

## Supplementary Materials

The following supporting information can be downloaded at: https://www.mdpi.com/article/10.3390/ijms26104469/s1.
