# Supplementary material for: Bioavailability for the Improved Therapeutic Profile of trans-Dehydrocrotonin Incorporated into a Copaiba Oil Self-Nanoemulsifying Drug Delivery System: Formulation, Physicochemical Characterizations, and Antioxidant In Vitro Effect
Source: Int J Mol Sci. 2025 May 8;26(10):4469. doi: 10.3390/ijms26104469 (PMC12110985; doi:10.3390/ijms26104469)

Supporting Information

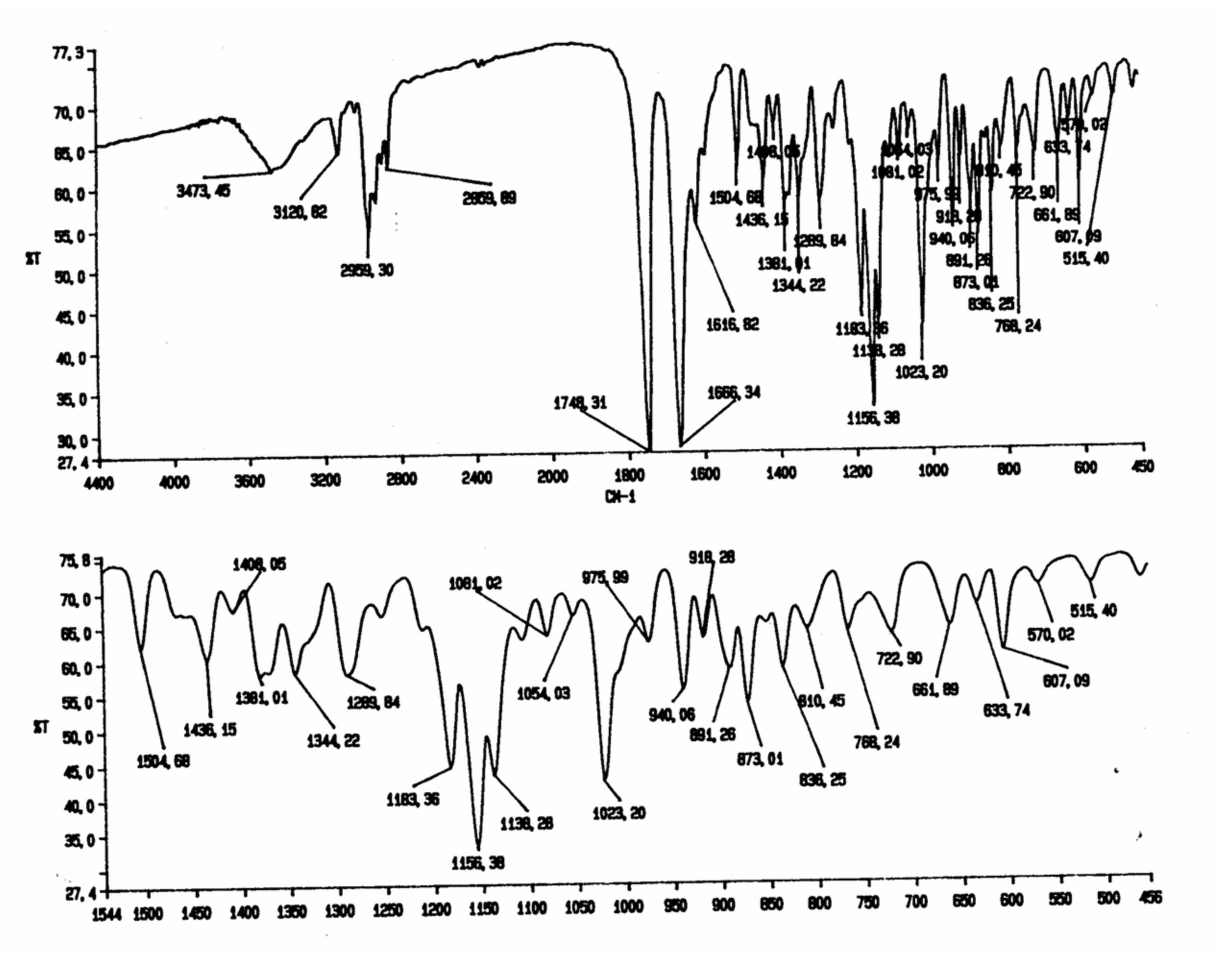

IR spectra of *trans*-dehydrocrotonin

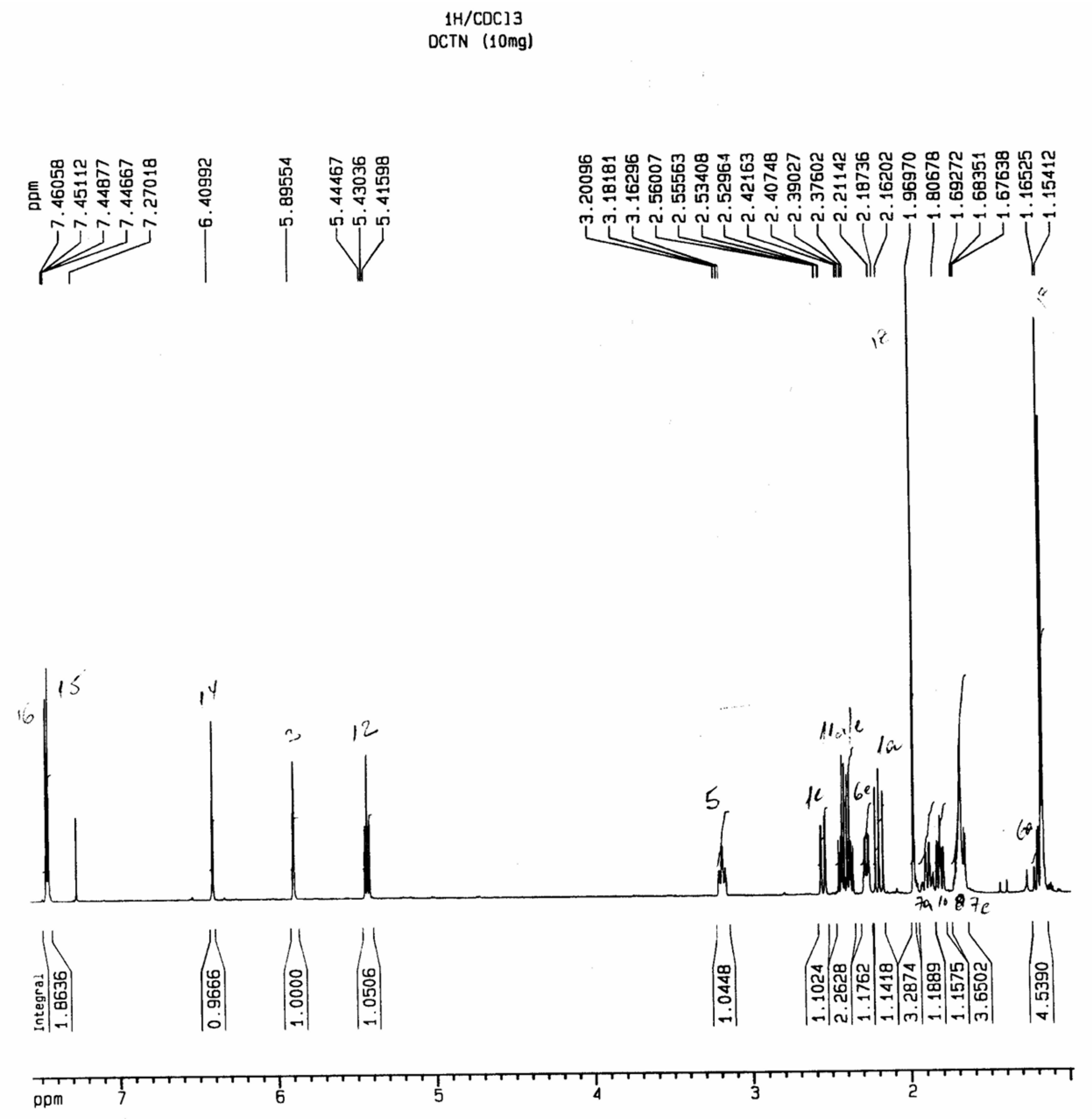

^{1}H NMR spectra of *trans*-dehydrocrotonin

1H/CDCl3
DCTN (10mg)

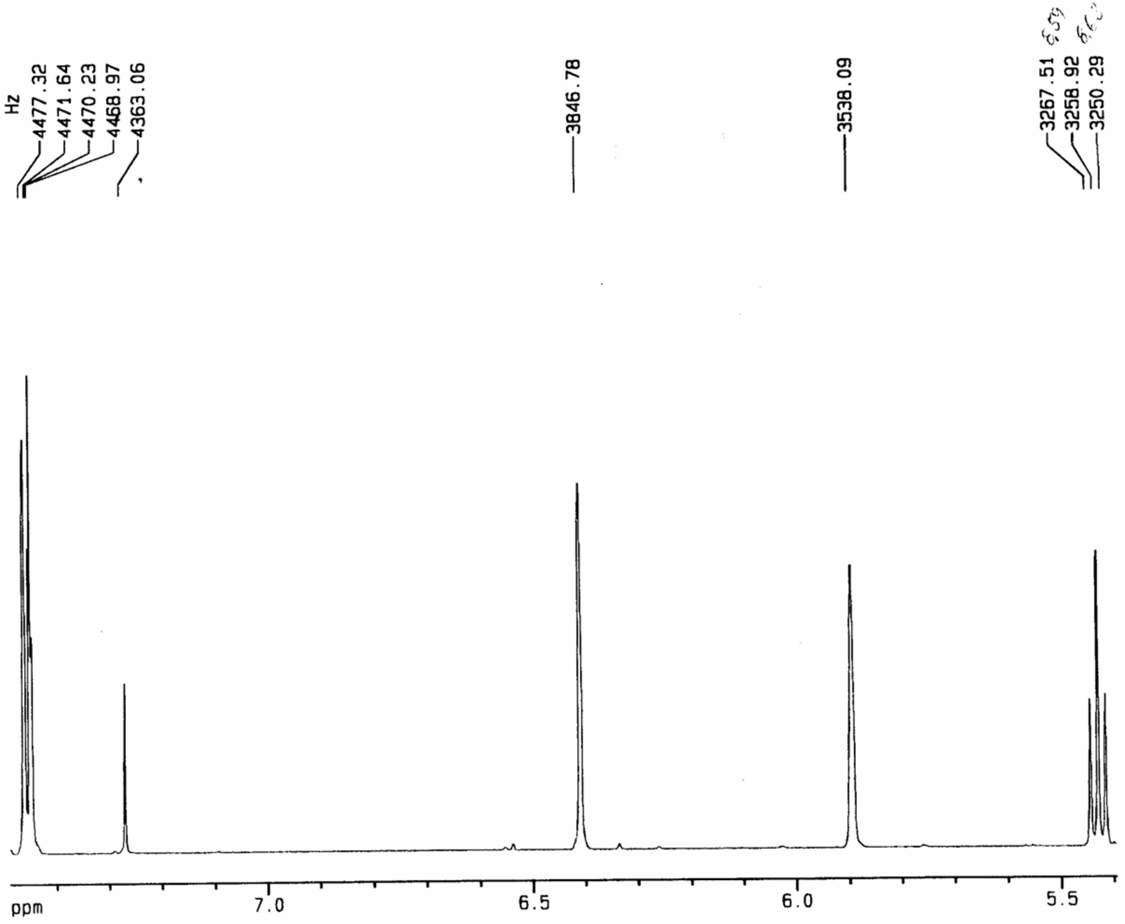

1H/CDCl3
DCTN (10mg)

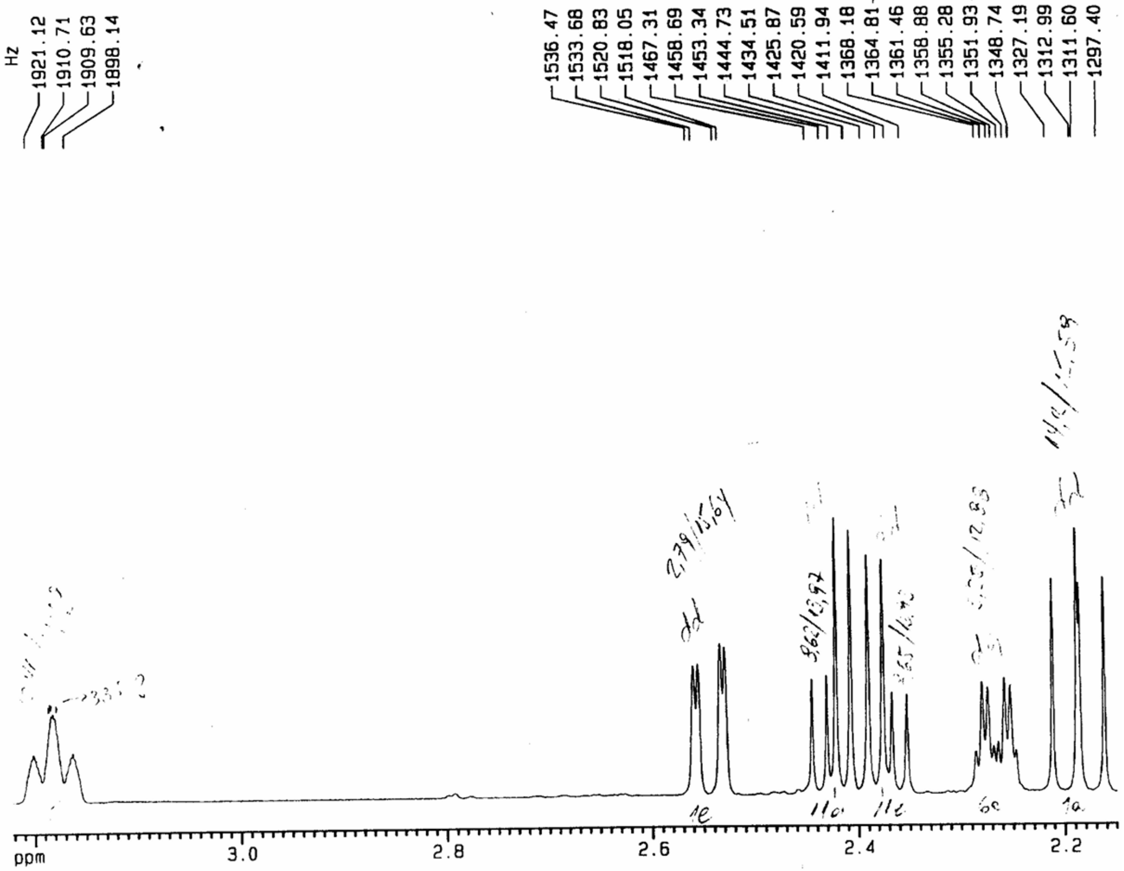

1H/CDCl3
DCTN (10mg)

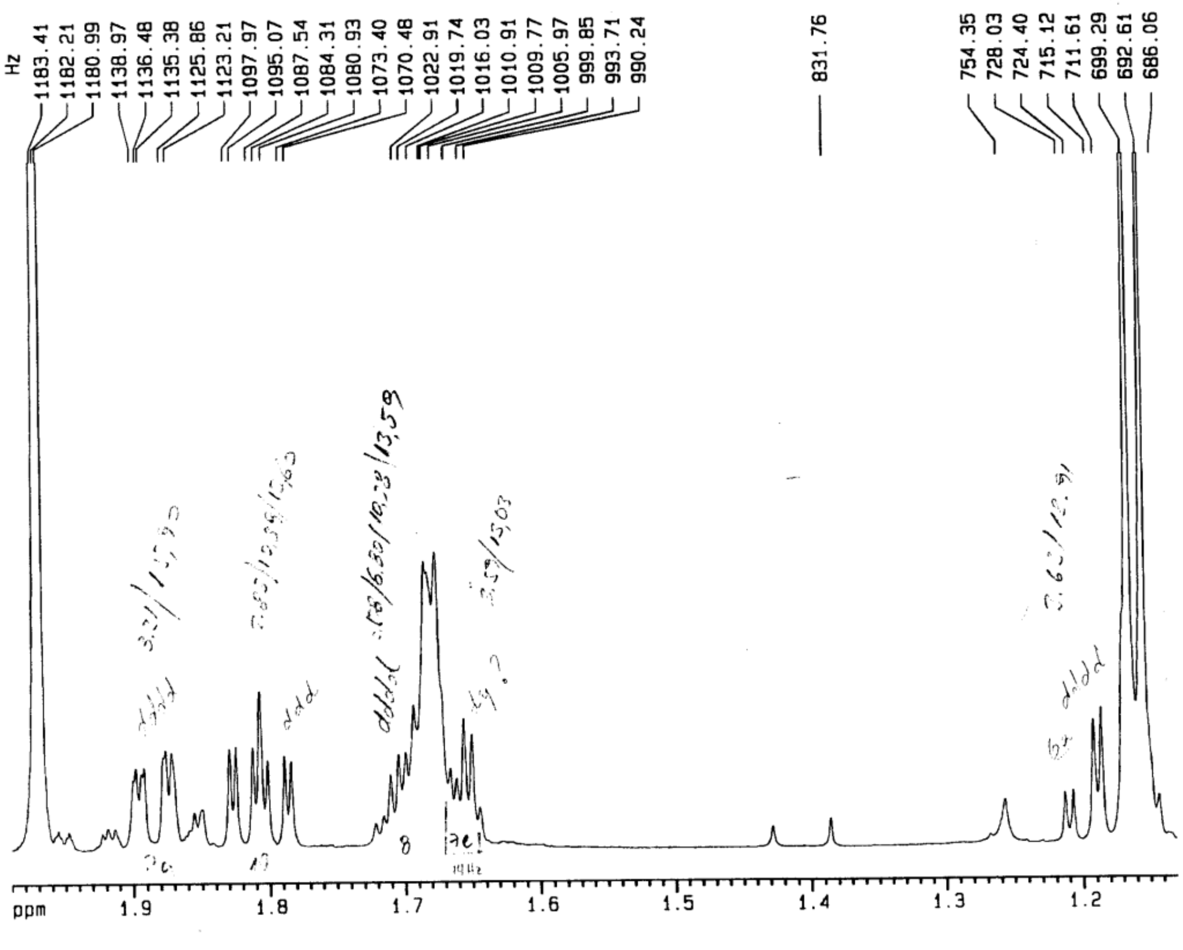

Supplement: Supplementary file 1 [file ijms-26-04469-s001.zip › ijms-3398666-supplementary.pdf]
